# Comparative Effectiveness of Published Interventions for Elderly Fall Prevention: A Systematic Review and Network Meta-Analysis

**DOI:** 10.3390/ijerph15030498

**Published:** 2018-03-12

**Authors:** Peixia Cheng, Liheng Tan, Peishan Ning, Li Li, Yuyan Gao, Yue Wu, David C. Schwebel, Haitao Chu, Huaiqiong Yin, Guoqing Hu

**Affiliations:** 1 Department of Epidemiology and Health Statistics, Xiangya School of Public Health, Central South University, 110 Xiangya Rd., Changsha 410078, China; chengpeixia92@163.com (P.C.); hersey.tan@foxmail.com (L.T.); ningpeishan@csu.edu.cn (P.N.); cslilee1009@gmail.com (L.L.); gaoyuyangyy@foxmail.com (Y.G.); 2 Department of Environmental and Occupational Health, Xiangya School of Public Health, Central South University, 110 Xiangya Rd., Changsha 410078, China; wuyue7802@csu.edu.cn; 3 Department of Psychology, University of Alabama, Birmingham, AL 35294, USA; schwebel@uab.edu; 4 Division of Biostatistics, The University of Minnesota, Minneapolis, MN 55455, USA; chux0051@umn.edu; 5 Central South University Library, 172 Tongzipo Rd., Changsha 410013, China; yinhuaiqionglib@mail.csu.edu.cn

**Keywords:** falls, elderly, prevention, network meta-analysis

## Abstract

Background: Falls are a major threat to older adults worldwide. Although various effective interventions have been developed, their comparative effectiveness remains unreported. Methods: A systematic review and network meta-analysis was conducted to determine the most effective interventions to prevent falls in community-dwelling adults aged 60 and over. Combined odds ratio (OR) and 95% credible interval (95% CrI) were calculated. Results: A total of 49 trials involving 27,740 participants and 9271 fallers were included. Compared to usual care, multifactorial interventions (MFI) demonstrated the greatest efficacy (OR: 0.64, 95% CrI: 0.53 to 0.77) followed by interventions combining education and exercise (EDU + EXC) (OR: 0.65, 95% CrI: 0.38 to 1.00) and interventions combining exercise and hazard assessment and modification (EXC + HAM) (OR: 0.66, 95% CrI: 0.40 to 1.04). The effect of medical care performed the worst (OR: 1.02, 95% CrI: 0.78 to 1.34). Model fit was good, inconsistency was low, and publication bias was considered absent. The overall quality of included trials was high. The pooled odds ratios and ranking probabilities remained relatively stable across all sensitivity analyses. Conclusions: MFI and exercise appear to be effective to reduce falls among older adults, and should be considered first as service delivery options. Further investigation is necessary to verify effectiveness and suitableness of the strategies to at-risk populations.

## 1. Introduction

Falls are a major cause of morbidity and mortality in elderly people [1,2]. Each year, about 20–35% of older adults aged 60 and over suffer a fall worldwide [3,4,5,6]. Approximately one in five falls leads to a serious injury, such as a traumatic brain injury or hip fracture, that is associated with high medical cost, emotional and physical pain, and substantial burden to the individual, family and society [7,8].

With the number of people living into older age rising rapidly, reducing and managing falls among the elderly has been a focus of research for decades [9]. Various interventions have been developed through randomized controlled trials (RCTs) and subsequently summarized in systematic reviews and meta-analyses [9,10,11,12,13,14]. Certain interventions such as exercise, home safety assessment and modification interventions, multifactorial interventions, and others have been demonstrated to be effective [15,16]. Unfortunately, most interventions have been assessed only in single randomized trials, limiting the ability to use traditional meta-analysis techniques to examine the comparative effectiveness of all published interventions.

Network meta-analysis (NMA), which combines data from randomized comparisons to deliver an internally consistent set of estimates while respecting the randomization in the evidence [17], offers an alternative analysis strategy. NMA overcomes the limitation of single studies examining each intervention by creating a graph of the network that summarizes the number of trials and studies, the number of participants studied in each trial, and a ranking of the effectiveness of published interventions even when they have been studied in only a single rigorous randomized trial [18,19].

This study was designed, therefore, to use NMA to evaluate the comparative effectiveness of all published elderly fall interventions and offer evidence to support effectiveness prioritization of interventions for elder fall prevention.

## 2. Materials and Methods

### 2.1. Search Strategy and Selection Criteria

We underwent searches of PubMed, Embase (Excerpta Medica Database), Cochrane Library, Web of Science, CINAHL (Cumulative Index to Nursing and Allied Health Literature), and four major Chinese databases (CMBdisc (China Biology Medicine disc), CNKI (China National Knowledge Infrastructure), WanFang Data Knowledge Service Platform and VIP Database for Chinese Technical Periodical) up to 31 December 2016 without any language restrictions, using search terms “fall*”; “aged”; “senior”; “elderly”; “older”. Supplementary Table S1 details the search strategy undertaken in PubMed as an example; similar language and syntax were used with the other databases.

Based on previously published studies [20,21,22], we classified fall prevention interventions into 11 categories: (1) usual care (no specific fall intervention), (2) education (EDU), (3) risk assessment and suggestion (RAS), (4) exercise (EXC), (5) medical care (MED), including vitamin D3 treatment, ophthalmology treatment, etc., (6) hazard assessment and modification (HAM), including personal or environmental safety recommendations and modification, (7) combination of education and RAS (EDU + RAS), (8) combination of education and exercise (EDU + EXC), (9) combination of RAS and exercise (RAS + EXC), (10) combination of exercise and HAM (EXC + HAM), and (11) multifactorial intervention (MFI), including three or more interventions listed above, such as the combination of EDU, RAS and EXC.

Studies identified from search databases were assessed by two independent authors according to the following eligibility criteria: (1) Study designed as randomized controlled trial; (2) targeted participants were limited to community-dwelling people, all of them aged 60 years or older; (3) fall was the primary outcome measure; (4) one or more categories of falls prevention interventions were studied, with at least one control group in the trial; eligible comparators included usual care, “placebo” control or unrelated interventions; and (5) publication on 31 December 2016 or earlier. Studies were excluded if targeted participants included people younger than 60 years of age, non-community (hospital, nursing home, or other long-term care facilities) residents, or were restricted to specialized populations (e.g., female or male alone, or stroke, osteoporosis, Parkinson disease patients). Studies were also excluded if they did not report fall-related outcomes or were reported only as abstracts. In addition, we excluded studies comparing the effectiveness of specific interventions within the same category mentioned above. For example, exercises included strength training, balance training, Tai Chi, community center based group exercise, home based exercise, etc., but we combined them into a single category “exercise” in this study and excluded papers that may have compared strength training to Tai Chi, for example.

Where two or more studies reported data from the same sample, only the latest one with detailed data was included in the analysis. The initial literature search was conducted by two reviewers (PXC and HQY) and then, PXC and LHT retrieved and independently screened full-text articles. Conflicts over inclusion were resolved through discussion. Data were extracted by one reviewer (PXC) and checked by LHT.

The review protocol was registered in the PROSPERO database (CRD42016027779) [23]. A PRISMA extension for NMA [24] was used to report this systematic review.

### 2.2. Data Analysis

Information was extracted from each included trial on: (1) Descriptive characteristics of study (first author, setting, published year, study time period, sample size); (2) sample characteristics (average age, gender percentage, and withdrawal rate); (3) inclusion and exclusion criteria of participants; (4) intervention and comparator characteristics (delivery and description); (5) fall-related outcome data (number of fallers, length of follow-up, effect of the intervention).

Following Cochrane Collaboration recommendations [25], we considered seven items to assess risk of bias: (1) Random sequence generation, (2) allocation concealment, (3) blinding of participants and personnel, (4) blinding of outcome assessment, (5) incomplete outcome data, (6) selective outcome reporting, and (7) other sources of bias. The risk of bias for each item was judged as one of three grades: low, unclear, or high. Conflicts were resolved by consensus.

We conducted both traditional pairwise meta-analysis and Bayesian NMA. Traditional pairwise meta-analysis was performed for comparisons reported in three studies or more using Review Manager (version 5.3, Cochrane Collaboration, Oxford, UK) software and the Mantel–Haenszel statistical method. We estimated effects of interventions by using pooled odds ratios (ORs) and their 95% confidence intervals. Pooled ORs, sometimes labeled as combined ORs, represent the effect size based on the information of all included studies in meta-analysis. Heterogeneity across studies was assessed with the *I*^2^ statistic, which measures the proportion of observed variance between trials that is due to real differences in effect size; *I*^2^ less than 25% was regarded as low and *I*^2^ greater than 75% as high [25]. Random-effect models [26] were used to address high heterogeneity across studies.

Contrasted-based NMA was performed to combine both direct and indirect evidence with the model proposed by Dias and colleagues [27] (chains = 1, iterations = 40,000 and burnin = 10,000) using WinBUGS (version 1.4.3, MRC Biostatistics Unit, Cambridge, UK). Pooled odds ratios and 95% credible intervals (95% CrIs; note: that 95% credible intervals are represented as 95% CrIs in NMA [27]) were calculated to quantify comparative effectiveness of interventions. In the Bayesian inference, the posterior distribution was calculated by combining information about prior distributions and observed data. We chose contrast-based NMA rather than arm-based NMA because a primary objective of this study was to evaluate relative effectiveness of available interventions [19,28].

To evaluate the interventions for fall prevention, we estimated the relative ranking probability of each intervention for its relative effectiveness using rankograms, mean rank and surface under the cumulative ranking curve (SUCRA) probabilities with Stata (version 12.0, StataCorporation, College Station, TX, USA). SUCRA is a percentage of the efficacy or safety of every intervention relative to an imaginary intervention that is always the best without uncertainty [29,30]. Thus, SUCRA equals 1 when an intervention is certain to be the best and 0 when an intervention is the worst [31].

The residual deviance was calculated to assess the goodness-of-fit of the model to the data, using the code proposed by Salanti [32] with R (version 3.2.3, R Foundation for Statistical Computing, Vienna, Austria). For an adequately fitting model, residual deviance is approximately equal to the unconstrained data points [33].

Inconsistent evidence is a sort of discrepancy that lies between direct evidence and indirect evidence [34]. We used three methods to assess the inconsistency between direct and indirect comparison. First, we compared the pooled ORs from NMA with corresponding ORs from traditional pairwise meta-analysis of direct comparison. Insignificant differences indicate consistency between direct and indirect comparisons. Second, we calculated inconsistency factor using the ifplot command [29] in Stata (version 12.0) to quantify the difference between direct and indirect evidence in all closed loops in the network. The simplest loop is a triangle formed by three direct comparison studies with shared comparators. A 95% CrI of a loop excluding zero indicates inconsistency between direct and indirect evidence [29,35]. Third, we investigated global inconsistency in the network using the “design-by-treatment” interaction model [36], which provides a single inference about the plausibility of assuming consistency throughout the entire network. Insignificant results indicate consistency between direct and indirect comparisons.

The extent of small study effects, publication bias and related bias were assessed by visual inspection of a funnel plot [26]. Publication bias was considered absent if the plot resembled a symmetrical inverted funnel. Sensitivity analysis was performed by excluding studies with two or more high-risk biases, studies with ≥5 “unclear” biases and studies with a follow-up time of <0.5 year.

### 2.3. Role of the Funding Sources

The funder of the study had no role in study design, data collection, data analysis, data interpretation, or writing of the report. The corresponding author had full access to all the data in the study and had final responsibility for the decision to submit for publication.

## 3. Results

**Figure 1:**
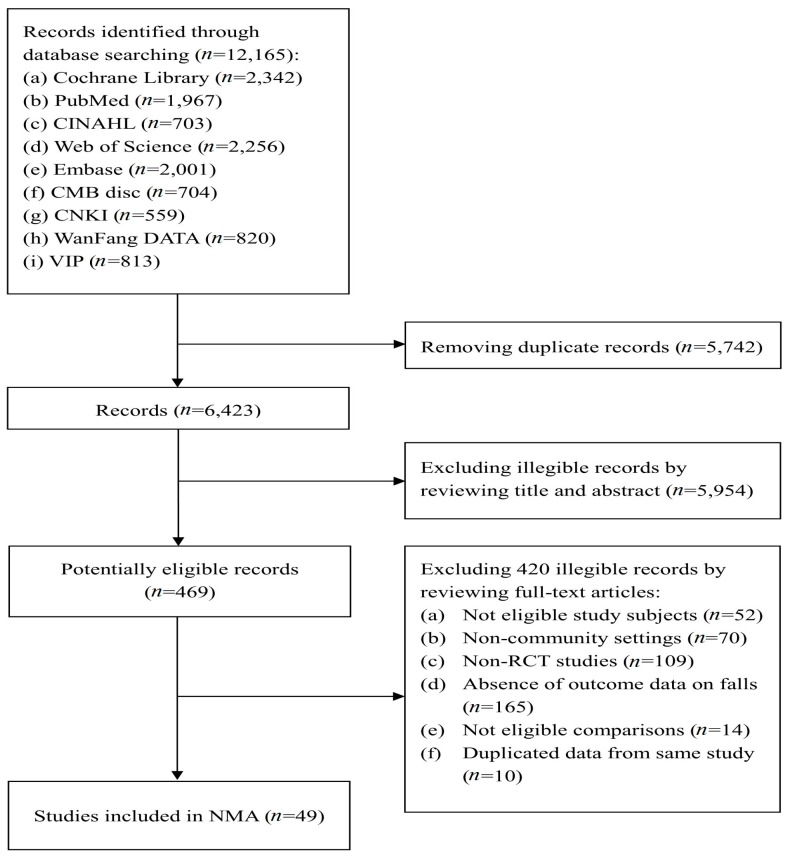
Flow diagram of study selection in systematic review of fall prevention interventions. Note: Abbreviations: 1. Embase: Excerpta Medica Database; 2. CINAHL: Cumulative Index to Nursing and Allied Health Literature; 3. CMBdisc: China Biology Medicine disc; 4. WanFang DATA: WanFang Data Knowledge Service Platform 5. VIP: VIP Database for Chinese Technical Periodical.

Our search strategy led to retrieval of, a total of 12,165 articles, of which 469 articles remained after removing duplicates and screening the titles and abstracts. After full-text assessment, 420 were excluded and 49 RCTs [36,37,38,39,40,41,42,43,44,45,46,47,48,49,50,51,52,53,54,55,56,57,58,59,60,61,62,63,64,65,66,67,68,69,70,71,72,73,74,75,76,77,78,79,80,81,82,83,84] involving 27,740 enrolled participants and 9271 fallers were included in the NMA (Figure 1). Study characteristics are summarized in Supplementary Table S2. The studies reported a mean follow-up period of 13.6 months (range: 4 to 60 months). The sample size in the individual trials ranged from 34 to 3182 participants, with a mean sample size of 578. Most studies (46 of 49) reported the mean ages of participants, which ranged from 67.5 to 88.0 (mean age: 73.0 years old) around 65% of participants were women.

Among the included studies, usual care was the most frequently investigated intervention. It was included in 35 studies. Seven direct comparisons were reported in three or more studies; these included MFI, EXC, MED, RAS and HAM versus usual care in 13, 11, 5, 3 and 3 studies, respectively, and MFI and EDU + EXC versus EDU in 7 and 4 studies, respectively (Supplementary Table S3 and Figure S1).

Risk of bias ratings for the included RCTs are presented in Supplementary Figures S2 and S3. Most included studies implemented random sequence generation (36 of 49) [36,37,39,41,42,43,44,45,47,48,49,50,51,52,53,54,56,58,60,61,62,64,65,66,69,70,71,72,73,74,75,76,78,82,83,84] and allocation concealment (35 of 49) [36,37,41,42,43,45,46,47,48,49,50,51,52,53,54,55,56,58,60,61,62,64,65,66,68,69,70,71,72,73,74,75,76,78,84]. Almost half of those studies (20 of 49) [38,42,43,48,49,51,53,55,58,62,63,66,69,70,72,73,74,75,77,79] had a low risk of bias in blinding of participants and personnel, and over half (29 of 49) [38,39,41,42,45,46,47,48,49,50,51,52,53,55,56,57,58,60,62,64,66,68,69,70,72,73,75,77,79] had a low risk of bias in blinding of outcome assessment. Due to a long follow-up period in most studies (mean: 13.6 months), attrition bias was common, and nine trials [37,43,53,60,63,70,74,76,80] had a high risk of bias in the incomplete outcome data category. For the selective reporting domain, 26 studies [37,38,39,41,42,44,45,46,47,48,50,51,52,53,54,55,60,63,64,68,69,70,72,73,75,76] were judged as having low risk of bias. Two studies [48,52] received commercial funding or lacked details of the interventions and were therefore judged as high risk of bias in the other bias category.

The NMA yielded pooled odds ratios for all possible comparisons among the 11 kinds of fall prevention interventions (Table 1). Compared to usual care, only RAS, EXC and MFI significantly reduced fall incidence, with respectively pooled odds ratios of 0.67 (95% CrI: 0.48 to 0.96), 0.67 (95% CrI: 0.52 to 0.84) and 0.64 (95% CrI: 0.53 to 0.77). MFI was also more effective than EDU (0.81, 95% CrI: 0.64 to 1.00) and MED (0.63, 95% CrI: 0.46 to 0.84). Despite large or moderate pooled odds ratios, some comparisons between interventions were not statistically significant because of inadequate sample size.

**Figure 2:**
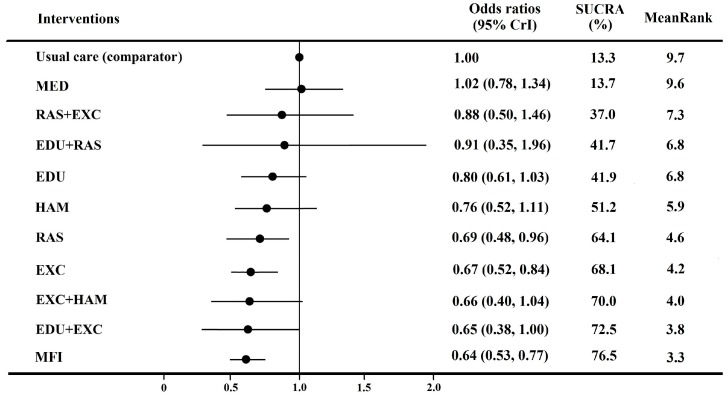
Pooled odds ratio, SUCRA and mean rank for each regimen, compared with usual care. Note: Labels of interventions: 1. Usual care (No specific fall intervention); 2. EDU (Education); 3. RAS (Risk assessment and suggestions); 4. EXC (Exercise); 5. MED (Medical care) 6. HAM (Hazard assessment and modification); 7. EDU + RAS (Education + risk assessment and suggestions); 8. EDU + EXC (Education + exercise); 9. RAS + EXC (Risk assessment and suggestions + exercise); 10. EXC + HAM (Exercise + hazard assessment and modification); 11. MFI (Multifactorial interventions). SUCRA (surface under the cumulative ranking curve probabilities) is a percentage of the efficacy or safety of every intervention relative to an imaginary intervention that is always the best without uncertainty. SUCRA = 1 means best intervention, worst SUCRA = 0).

According to the ranked orders in SUCRA, MFI had the largest SUCRA (76.5%) and ranked first (mean rank: 3.3). EDU + EXC, EXC + HAM and EXC ranked second, third and fourth, respectively (Figure 2, Figures S4 and S5). Usual care and MED had the lowest mean ranks (9.7 and 9.6).

For all comparisons reported in three studies or more, the results from the traditional pairwise meta-analysis and Bayesian NMA generally showed consistent results (Supplementary Figure S6).

The residual deviance of NMA models closely approached the unconstrained data points (101.88 versus 104) (Supplementary Figure S7). Consistency between results from traditional pairwise meta-analysis and NMA was assessed through three methods. First, we compared the pooled odds ratios from NMA with corresponding odds ratios from traditional pairwise random effects meta-analysis. The two methods yielded similar pooled odds ratios (Supplementary Figure S6). Second, we conducted inconsistency analysis using loop-specific heterogeneity estimate and found consistent loops for all closed loops (95% CIs of all loops were truncated at zero) (Supplementary Figure S8). Third, we ran a “design-by-treatment” interaction model to evaluate global inconsistency in network; the interaction was not significant (*p* = 0.974).

Multiple intervention comparisons showed a roughly symmetrical inverted funnel (Supplementary Figure S9). We also performed sensitivity analysis to evaluate the impact both of studies with high-risk biases and with various follow-up periods on our results. The pooled odds ratios and ranking probabilities for the 11 kinds of interventions remained relatively stable across sensitivity analyses with some minor changes in most cases, which suggests robustness of results to publications with ≥2 high-risk biases [36,37,43,74,76] and short follow-up periods of <6 months [36,58,67] (Table 2 and Table S4). However, the ranks of 11 types of fall interventions changed greatly when we omitted the results of all eight studies with ≥5 unclear biases [40,59,67,80,81,82,83,84].

## 4. Discussion

In this NMA, we considered 49 RCTs comparing the relative efficacy of 11 kinds of interventions for elderly fall prevention. We generated several noteworthy findings. First, MFI was the most effective intervention for falls, followed by EDU + EXC, EXC + HAM, and EXC. Second, RAS and EXC achieved nearly the same effectiveness compared to MFI, with respective ORs of 0.95 (95% CrI: 0.64 to 1.39) and 0.97 (95% CrI: 0.73 to 1.30), although their ranks were somewhat lower.

Most included studies in this NMA had low-risk bias. Also, NMA models fit the included data well and Loop-specific heterogeneity estimate and “design-by-treatment” interaction models both showed consistent evidence across direct and indirect comparisons. In addition, sensitivity analyses indicate that the findings are robust to studies with high-risk biases and short follow-up time periods. These results support the validity and creditability of our findings.

Although a number of systematic reviews have synthesized findings from single or multifactorial fall prevention interventions [9,10,11,12,13], most focused on studies directly comparing a specific fall prevention intervention with usual care. Our study used NMA to evaluate comparative effectiveness of 11 types of published fall interventions, with five kinds of interventions emerging as the most effective: MFI, EDU + EXC, EXC + HAM, EXC and RAS. These results generally concord with previous results from traditional meta-analysis [10,11,12,13,14].

Notably, although MFI ranked as the most effective intervention in study, its comparative advantage was not obvious compared to single interventions “EXC” and “RAS”. This replicates the findings of Campbell and Robertson [13] that single interventions might be as effective in reducing falls as MFI. When prevention resources are limited, single interventions like EXC and RAS should be considered.

The fact that MFI was more effective than most single interventions may be due to one of three possibilities: (a) It includes components of single interventions that are effective, and/or (b) the interaction between two or more components of MFI create positive results, and/or (c) different interventions are effective for different individuals based on any number of individual differences, and applying MFI increases the likelihood of a “match” between an effective intervention and particular individuals. Our data did not permit further analyses to explore these or other hypotheses, and the contents and implementation details of MFI vary widely across studies, although we recommend future research to investigate these topics.

Our study replicates the findings of a Cochrane systematic review and meta-analysis that included 159 trials with 79,193 participants and concluded that vitamin D did not reduce fall rates (1.00, 95% CrI 0.90 to 1.11; 7 trials; 9324 participants) for healthy older people, that interventions to treat vision problems may result in a significant increase in the rate of falls (1.57, 95% CrI 1.19 to 2.06; 616 participants), and that education alone did not significantly reduce the rate of falls (0.33, 95% CrI 0.09 to 1.20; 1 trial; 45 participants) [23]. Our finding that MFI might reduce the risk of injurious falls when compared with usual care also concords with recent results from a traditional meta-analysis by Tricco et al. [10].

Our results represent the first quantification of the comparative effectiveness of several interventions, including MFI versus RAS (0.97, 95% CrI: 0.73 to 1.30), MFI versus. EXC + HAM (1.03, 95% CrI: 0.60 to 1.66), MFI versus MED (0.63, 95% CrI: 0.46 to 0.84), and MFI versus HAM (0.87, 95% CrI: 0.55 to 1.25). Although several of those indirect comparisons were insignificant because of small sample sizes, almost all the pooled odds ratios deviated from 1.0, offering suggestive insights on the best fall prevention interventions.

Potential limitations of our study should be noted. First, the number of studies included for certain interventions is limited, leading to wide and insignificant 95% CrIs of pooled ORs in NMAs even when pooled ORs far deviated from 1.0. Second, we did not quantify the comparative effectiveness of specific interventions within the same category due to an inadequate number of published studies. For example, we combined all types of exercises into a single category of “exercise” because many interventions were reported in only one study. In addition, we combined all studies with three or more interventions as “multifactorial” (MFI) and did not distinguish between those with three factors vs. four or five or more factors. Last, we did not assess fall-induced morbidity, mortality or cost because of inadequate data.

## 5. Conclusions

In conclusion, our study offers implications for both practitioners and researchers. For practitioners, the ranking of the ten kinds of interventions to reduce falls among older adults might guide selection of interventions. Efficacy will need to be balanced with available resources, but the interventions that emerged with the highest ranks in our analysis—MFI, EDU + EXC, EXC + HAM, EXC and RAS—might be considered in practice when resources allow. For researchers, our study emphasizes the value to update meta-analyses and NMA on this topic regularly, as new data are constantly being published. Further investigation is necessary to verify effectiveness and suitableness of the strategies to at-risk or specialized populations. In addition, large-scale trials to compare the effectiveness of the top several interventions in our rankings are needed to differentiate their efficacy in the same randomized sample.

## Figures and Tables

**Table 1:** Results of pooled odds ratios (95% CrI) for fall prevention interventions using Bayesian Hazard assessment and modification (NMA).

**(1) Usual Care**	0.80 (0.61, 1.03)	0.69 (0.48, 0.96) *	0.67 (0.52, 0.84) *	1.02 (0.78, 1.34)	0.76 (0.52, 1.11)	0.91 (0.35, 1.96)	0.65 (0.38, 1.00)	0.88 (0.50, 1.46)	0.66 (0.40, 1.04)	0.64 (0.53, 0.77) *
	**(2) Education (EDU)**	0.88 (0.56, 1.33)	0.85 (0.60, 1.12)	1.30 (0.89, 1.80)	0.97 (0.63, 1.51)	1.14 (0.48, 2.40)	0.81 (0.50, 1.21)	1.12 (0.60, 2.04)	0.84 (0.48, 1.40)	0.81 (0.64, 1.00) *
		**(3) Risk assessment and suggestions (RAS)**	1.00 (0.65, 1.50)	1.53 (0.98, 2.32)	1.14 (0.65, 1.80)	1.37 (0.53, 2.97)	0.96 (0.50, 1.74)	1.32 (0.67, 2.38)	0.98 (0.52, 1.67)	0.95 (0.64, 1.39)
			**(4) Exercise (EXC)**	1.55 (1.10, 2.18)	1.16 (0.74, 1.76)	1.38 (0.55, 3.07)	0.98 (0.56, 1.57)	1.33 (0.75, 2.29)	0.99 (0.59, 1.57)	0.97 (0.73, 1.30)
				(**5) Medical care (MED)**	0.76 (0.48, 1.14)	0.91 (0.35, 2.03)	0.64 (0.35, 1.06)	0.88 (0.46, 1.51)	0.65 (0.38, 1.07)	0.63 (0.46, 0.84) *
					**(6) Hazard assessment and modification (HAM)**	1.23 (0.43, 2.70)	0.88 (0.44, 1.48)	1.20 (0.57, 2.16)	0.89 (0.48, 1.51)	0.87 (0.55, 1.25)
						**(7) EDU + RAS**	0.83 (0.30, 1.92)	1.16 (0.39, 2.69)	0.86 (0.30, 1.94)	0.83 (0.33, 1.76)
							**(8) EDU + EXC**	1.46 (0.63, 2.98)	1.09 (0.52, 2.12)	1.04 (0.64, 1.66)
								**(9) RAS + EXC**	0.81 (0.37, 1.56)	0.78 (0.42, 1.32)
									**(10) EXC + HAM**	1.03 (0.60, 1.66)
										**(11) Multifactorial interventions (MFI)**

Notes: Pooled odds ratios represent the effect size based on the information of all included studies in meta-analysis, which between two comparator groups were calculated using the first group as the reference. 95% CrIs means 95% credible intervals in NMA. Labels of interventions: 1. Usual care (Namely without any specific fall intervention); 2. EDU (Education); 3. RAS (Risk assessment and suggestions); 4. EXC (Exercise); 5. MED (Medical care) 6. HAM (Hazard assessment and modification); 7. EDU + RAS (Education + risk assessment and suggestions); 8. EDU + EXC (Education + exercise); 9. RAS + EXC (Risk assessment and suggestions + exercise); 10. EXC + HAM (Exercise + hazard assessment and modification); 11. MFI (Multifactorial interventions). * *p* < 0.05.

**Table 2:** Changes in pooled odds ratios of ten comparator groups in comparison to usual care after excluding studies with high-risk biases or unclear biases.

Analysis Strategies	EDU	RAS	EXC	MED	HAM	EDU + RAS	EDU + EXC	RAS + EXC	EXC + HAM	MFI
All 49 studies	0.80 (0.61, 1.03)	0.69 (0.48, 0.96) *	0.67 (0.52, 0.84) *	1.02 (0.78, 1.34)	0.76 (0.52, 1.11)	0.91 (0.35, 1.96)	0.65 (0.38, 1.00)	0.88 (0.50, 1.46)	0.66 (0.40, 1.04)	0.64 (0.53, 0.77) *
Exclude Ref. [36]	0.80 (0.61, 1.02)	0.68 (0.47, 0.96) *	0.68 (0.54, 0.85) *	1.02 (0.76, 1.36)	0.76 (0.50, 1.10)	0.89 (0.35, 1.85)	0.65 (0.39, 1.06)	0.90 (0.48, 1.54)	0.65 (0.38, 1.02)	0.64 (0.53, 0.77) *
Exclude Ref. [37]	0.80 (0.60, 1.04)	0.69 (0.47, 0.95) *	0.67 (0.53, 0.82) *	1.02 (0.75, 1.35)	0.77 (0.51, 1.14)	0.92 (0.35, 2.00)	0.66 (0.36, 1.06)	0.89 (0.48, 1.56)	0.65 (0.39, 1.04)	0.64 (0.52, 0.76) *
Exclude Ref. [43]	0.80 (0.61, 1.03)	0.82 (0.53, 1.20)	0.67 (0.54, 0.82) *	1.02 (0.79, 1.28)	0.76 (0.52, 1.05)	0.91 (0.37, 1.95)	0.66 (0.39, 1.07)	0.91 (0.49, 1.51)	0.66 (0.39, 1.04)	0.64 (0.53, 0.78) *
Exclude Ref. [74]	0.80 (0.60, 1.03)	0.69 (0.49, 0.96) *	0.69 (0.54, 0.84) *	1.03 (0.78, 1.34)	0.77 (0.53, 1.08)	0.92 (0.37, 1.90)	0.64 (0.39, 1.06)	0.90 (0.48, 1.54)	0.66 (0.39, 1.05)	0.64 (0.52, 0.77) *
Exclude Ref. [76]	0.79 (0.60, 1.04)	0.69 (0.48, 0.97) *	0.65 (0.51, 0.82) *	1.01 (0.75, 1.33)	0.77 (0.52, 1.10)	0.90 (0.37, 1.89)	0.64 (0.38, 1.02)	0.88 (0.47, 1.50)	0.66 (0.39, 1.03)	0.64 (0.53, 0.76) *
Exclude Refs. A	0.80 (0.61, 1.05)	0.81 (0.51, 1.21)	0.68 (0.54, 0.87) *	1.02 (0.77, 1.32)	0.76 (0.52, 1.10)	0.91 (0.36, 1.90)	0.66 (0.39, 1.08)	0.88 (0.48, 1.43)	0.65 (0.38, 1.01)	0.65 (0.54, 0.78) *
Exclude Refs. B	0.90 (0.70, 1.14)	0.71 (0.53, 0.92) *	0.71 (0.60, 0.87) *	1.04 (0.81, 1.27)	0.78 (0.58, 1.06)	0.97 (0.44, 1.88)	0.75 (0.46, 1.12)	0.89 (0.55, 1.39)	0.68 (0.46, 1.02)	0.77 (0.63, 0.92) *
Exclude Refs. C	0.79 (0.62, 1.02)	0.68 (0.48, 0.94) *	0.68 (0.54, 0.84) *	1.01 (0.75, 1.35)	0.76 (0.50, 1.09)	0.87 (0.36, 1.84)	0.72 (0.40, 1.18)	0.90 (0.49, 1.52)	0.66 (0.38, 1.06)	0.64 (0.53, 0.77) *

Notes: Labels of interventions: 1. EDU (Education); 2. RAS (Risk assessment and suggestions); 3. EXC (Exercise); 4. MED (Medical care) 5. HAM (Hazard assessment and modification); 6. EDU + RAS (Education + risk assessment and suggestions); 7. EDU + EXC (Education + exercise); 8. RAS + EXC (Risk assessment and suggestions + exercise); 9. EXC + HAM (Exercise + hazard assessment and modification); 10. MFI (Multifactorial interventions). Refs. A: references [36,37,43,74,76] had ≥2 high-risk biases. Refs B: references [40,59,67,80,81,82,83,84] had ≥5 unclear biases. Refs. C: references [36,58,67] had a follow-up period of <6 months. * *p* < 0.05.

## Supplementary Materials

The following are available online at http://www.mdpi.com/1660-4601/15/3/498/s1, Figure S1: Network of interventions to prevent falls in the elderly. Figure S2: Judgments of risk for 7 items of bias in all 49 included studies. Figure S3: Risk summary of 49 studies included in NMA by item and article. Figure S4: Ranking of intervention strategies based on probability of their effects on outcome of falls (Rankogram). Figure S5: Surface under the cumulative ranking curve (SUCRA) probabilities diagram. Figure S6: Pooled odds ratios for fall incidence by Bayesian NMA and pairwise meta-analysis. Figure S7: Benchmark effect diagram. Figure S8: Inconsistency analysis using loop-specific heterogeneity estimate. Figure S9: Comparison-adjusted funnel plot. Table S1: Search strategy used for PubMed. Table S2: Characteristics of studies included in the NMA. Table S3: Contribution of direct evidence to the network. Table S4: Changes in rank based on SUCRA and Mean rank of 11 comparator groups after excluding studies with high-risk biases or unclear bias.
