# Supplementary material for: Comparative Effectiveness of Published Interventions for Elderly Fall Prevention: A Systematic Review and Network Meta-Analysis
Source: Int J Environ Res Public Health. 2018 Mar 12;15(3):498. doi: 10.3390/ijerph15030498 (PMC5877043; doi:10.3390/ijerph15030498)
Supplement: Supplementary file 1 [file ijerph-15-00498-s001.pdf]

Supplementary Materials

Table S1: Search strategy used for PubMed

No.	Search strategy
1	Accidental falls.mh.
2	Fall*.ti,ab.
3	1 or 2
4	(Aged* or senior* or elderly or older).ti,ab
5	Randomized controlled trial.pt.
6	Controlled clinical trial.pt.
7	Randomized.ti,ab.
8	Placebo.ti,ab.
9	Clinical trials as topic[mesh:noexp]
10	Randomly.ti,ab.
11	Trial.ti.
12	Animals.sh.
13	Humans.sh
14	12 not 13
15	5 or 6 or 7 or 8 or 9 or 10 or 11 not 14
16	3 and 4 and 15
17	Limit 16 to py=" initial to December 31, 2016"

Table S2: Characteristics of studies included in the network meta-analysis

First author, Year	Setting	Comparisons	Follow-up Period (months)	No. of Participants	No. of Fallers	Mean Age (years)	Female (%)	Participants' Criteria
Ansai JH, 2015 [1]	Brazil	Usual care vs EXC	5.5	69	28	82.4	68%	(1)⩾ 80, (2) sedentary, (3) able to walk alone
Barker AL, 2015 [2]	Australia	EDU vs EDU+EXC	6	53	15	69.3	88%	(1)⩾ 60, (2) negotiate a set of 10 stairs independently
Bischoff-Ferrari HA, 2006 [3]	USA	Usual care vs MED	36	445	231	70.8	55%	(1)⩾ 65
Blalock SJ, 2010 [4]	USA	EDU vs EDU+RAS	12	186	105	74.8	71%	(1)⩾ 65, (2)⩾ 1 fall in the past year, (3) ⩾ 4 chronic prescription medications (CNS-active medication ⩾ 1)
Cai Y, 2014 [5]	China	Usual care vs MFI	12	326	38	73.1	56%	(1)⩾ 60
Clemson L, 2004 [6]	Australia	Usual care vs MFI	14	310	171	78.4	74%	(1)⩾ 70, (2)⩾ 1 fall in the past year or concerned about falling
Clemson L, 2010 [7]	Australia	Usual care vs EXC	6	34	17	81.5	47%	(1)⩾ 70, (2)⩾ 2 falls or an injurious fall in the past year
Close J, 1999 [8]	UK	Usual care vs RAS	12	397	170	78.2	67%	(1)⩾ 65, (2) attended emergency department with 1st diagnosis of a fall
Cornillon E, 2002 [9]	France	Usual care vs MFI	12	303	87	71.1	83%	(1)⩾ 65, (2) MMSE* ⩾20, (3) walk at least 6 meters
Cumming RG, 1999 [10]	Australia	Usual care vs HAM	12	530	215	76.8	57%	(1)⩾ 65, (2)community-dwelling within study area
Cumming RG, 2007 [11]	Australia	Usual care vs MED	12	616	354	80.6	68%	(1)⩾ 70
Dorresteijn TA, 2016 [12]	Netherlands	Usual care vs EDU	12	389	200	78.3	70%	(1)⩾ 70, (2) reported at least some concerns about falls
Dukas L, 2004 [13]	Switzerland	Usual care vs MED	9	378	86	75.0	53%	(1)⩾ 70, (2) mobile, (3) independent life style
Elley CR, 2008 [14]	New Zealand	Usual care vs MFI	12	312	204	80.8	69%	(1)⩾ 75, (2)⩾ 1 fall in the past year
Fairhall N, 2014 [15]	Australia	Usual care vs MFI	12	241	139	83.3	68%	(1)⩾ 70, (2)MMSE* >18, (3) life expectancy⩾ 1 year
Fizharris MP, 2010 [16]	Australia	Usual care vs EXC vs MED vs HAM vs EXC+HAM vs MFI	18	1090	606	76.1	60%	(1)⩾ 70
Freiberger E, 2007 [17]	Germany	Usual care vs EXC	12	217	85	75.9	45%	(1)⩾ 70
Gawler S, 2016 [18]	UK	Usual care vs EXC	24	1256	334	73.0	62%	(1) ⩾ 65, (2) physically able to attend group exercise
Gianoudis J, 2014 [19]	Australia	EDU vs MFI	12	162	54	67.5	79%	(1)⩾ 60
Helbostad JL, 2004 [20]	Norway	EXC vs RAS+EXC	12	77	38	81.1	81%	(1)⩾ 75, (2)⩾ 1 fall in the past year or use of some kind of walking aid
Hogan DB, 2001 [21]	Canada	Usual care vs RAS	12	163	115	77.7	72%	(1) ⩾ 65, (2) ⩾ 1 fall in the past 3 months

Hornbrook MC, 1994 [22]	USA	EDU vs MFI	23	3182	1319	73.2	62%	(1)⩾ 65
Huang TT, 2011 [23]	Taiwan	EDU vs EDU+EXC	5	186	19	NR	59%	(1)⩾ 60
Jin X, 2009 [24]	China	EDU vs MFI	12	1703	107	73.2	59%	(1)⩾ 60
Logghe IH, 2009 [25]	Netherlands	EDU vs EDU+EXC	12	269	117	77.2	71%	(1)⩾ 70, (2) having a high fall risk
Lord SR, 2005 [26]	Australia	Usual care vs RAS vs MFI	12	620	90	80.4	66%	(1)⩾ 75, (2) low score on physiological profile assessment
Luukinen H, 2007 [27]	Finland	Usual care vs RAS+EXC	16	486	262	88.0	79%	(1)⩾ 85 (2)⩾ 1 risk factor for falling
McMurdo ME, 2000 [28]	UK	Usual care vs EXC+HAM	12	133	42	84.0	81%	(1)⩾ 70
Palvanen M, 2014 [29]	Finland	EDU vs MFI	12	1314	645	77.6	86%	(1)⩾ 70, (2)⩾ 1 risk factor for falling
Perula LA, 2012 [30]	Spain	EDU vs MFI	12	404	87	76.4	53%	(1)⩾ 70, (2) walk independently
Pighills AC, 2011 [31]	UK	Usual care vs HAM	12	238	154	79.0	67%	(1)⩾ 70, (2)⩾ 1 fall in the past year
Robson E, 2003 [32]	Canada	Usual care vs MFI	4	471	96	73.0	81%	(1)⩾ 65, (2) walk ⩾20 minutes and get down and off floor independently
Salminen MJ, 2009 [33]	Finland	EDU vs MFI	12	591	271	NR	84%	(1)⩾ 65, (2)⩾ 1 fall in the past year, (3) MMSE* ⩾17, (4) walk ⩾ 10m independently
Shumway-Cook A, 2007 [34]	USA	EDU vs EDU+EXC	12	453	254	75.6	77%	(1)⩾ 65, (2) primary care physician visit ⩾ 1 in the past 3 years, (3) no regular exercise in the past 3 months
Siegrist M, 2016 [35]	Germany	Usual care vs EXC	12	378	143	78.0	75%	(1)⩾ 65, (2) increased physical fall risk
Spice CL, 2009 [36]	UK	Usual care vs MFI	12	516	409	82.2	72%	(1)⩾ 65, (2)⩾ 2 falls in the past year
Tinetti ME, 1994 [37]	USA	Usual care vs MFI	12	301	120	77.9	69%	(1)⩾ 70,(2) ambulation (3) MMSE* ⩾20
Trivedi DP, 2003 [38]	UK	Usual care vs MED	60	2686	515	74.0	24%	(1)⩾ 65
Trombetti A, 2011 [39]	Switzerland	Usual care vs EXC	12	134	51	75.5	96%	(1)⩾ 65, (2) at increased risk of falling
Voukelatos A, 2007 [40]	Australia	Usual care vs EXC	6	702	152	69.0	84%	(1)⩾ 60, (2) had not practiced tai chi in the past year
Voukelatos A, 2015 [41]	Australia	Usual care vs EXC	12	386	122	73.2	74%	(1)⩾ 65, (2) inactive, mobile
Wagner EH, 1994 [42]	USA	Usual care vs RAS vs MFI	24	1559	469	72.5	59%	(1)⩾ 65, (2) ambulatory, (3) independent in activities of daily living
Weerdesteyn V, 2006 [43]	Netherlands	Usual care vs EXC	7	113	40	73.9	79%	(1)⩾ 65, (2)⩾ 1 fall in the past year, (3) walk independently ⩾ 15 min
Wolf SL, 2003 [44]	USA	EDU vs EXC	12	311	154	80.9	94%	(1)⩾ 70, (2) transitioning to frailty
Wu C, 2010 [45]	China	Usual care vs MFI	12	248	27	69.9	77%	(1)⩾ 60

Xia Q, 2010 [46]	China	Usual care vs MFI	12	2310	227	72.1	53%	(1)⩾ 60
Xie X, 2016 [47]	China	EDU vs MFI	6	280	29	NR	NR	(1)⩾ 65
Zhan J, 2013 [48]	China	Usual care vs EDU	12	112	33	75.4	42%	(1)61~90
Zhan P, 2010 [49]	China	Usual care vs EXC	12	100	25	67.6	39%	(1)⩾ 65

Notes: MMSE means mini-mental state examination score; NR means not reported. USA means United States of America. UK means United Kingdom.

Labels of interventions: 1. Usual care (Namely without any specific fall intervention); 2. EDU (Education); 3. RAS (Risk assessment and suggestions); 4. EXC (Exercise); 5. MED (Medical care) 6. HAM (Hazard assessment and modification); 7. EDU+RAS (Education + risk assessment and suggestions); 8. EDU+EXC (Education + exercise); 9. RAS+EXC (Risk assessment and suggestions + exercise); 10. EXC+HAM (Exercise + hazard assessment and modification); 11. MFI (Multifactorial interventions).

Table S3: Contribution of direct evidence to the network

No.	Comparison	Number of trials	Contribution to the network (%)
1	Usual care vs. MFI	10	20.41
2	Usual care vs. EDU	2	4.08
3	Usual care vs. RAS	2	4.08
4	Usual care vs. RAS vs. MFI	2	4.08
5	Usual care vs. EXC	10	20.41
6	Usual care vs. EXC vs. MED HAM vs. EXC+HAM vs. MFI	1	2.04
7	Usual care vs. MED	4	8.16
8	Usual care vs. HAM	2	4.08
9	Usual care vs. RAS+EXC	1	2.04
10	Usual care vs. EXC+HAM	1	2.04
11	EDU vs. MFI	7	14.29
12	EDU vs. EXC	1	2.04
13	EDU vs. EDU+RAS	1	2.04
14	EDU vs. EDU+EXC	4	8.16
15	EXC vs. RAS+EXC	1	2.04

Labels of interventions: 1. Usual care (Namely without any specific fall intervention); 2. EDU (Education); 3. RAS (Risk assessment and suggestions); 4. EXC (Exercise); 5. MED (Medical care) 6. HAM (Hazard assessment and modification); 7. EDU+RAS (Education + risk assessment and suggestions); 8. EDU+EXC (Education + exercise); 9. RAS+EXC (Risk assessment and suggestions + exercise); 10. EXC+HAM (Exercise + hazard assessment and modification); 11. MFI (Multifactorial interventions).

Table S4: Changes in rank based on SUCRA and Mean rank of 11 comparator groups after excluding studies with high-risk biases or unclear bias

Analysis strategies	Usual Care	EDU	RAS	EXC	MED	HAM	EDU+RAS	EDU+EXC	RAS+EXC	EXC+HAM	MFI
All 49 studies	11	7	5	4	10	6	8	2	9	3	1
Exclude Ref [1]	11	8	4	5	10	6	7	2	9	3	1
Exclude Ref [2]	11	7	5	4	10	6	8	3	9	2	1
Exclude Ref [8]	11	6	7	4	10	5	8	2	9	3	1
Exclude Ref [39]	11	7	5	4	10	6	8	2	9	3	1
Exclude Ref [41]	11	7	5	3	10	6	8	2	9	4	1
Exclude Refs A	11	7	6	4	10	5	8	3	9	2	1
Exclude Refs B	10	9	3	2	11	6	8	4	7	1	5
Exclude Refs C	11	8	4	3	10	6	7	5	9	2	1

Labels of interventions: 1. Usual care (Namely without any specific fall intervention); 2. EDU (Education); 3. RAS (Risk assessment and suggestions); 4. EXC (Exercise); 5. MED (Medical care) 6. HAM (Hazard assessment and modification); 7. EDU+RAS (Education + risk assessment and suggestions); 8. EDU+EXC (Education + exercise); 9. RAS+EXC (Risk assessment and suggestions + exercise); 10. EXC+HAM (Exercise + hazard assessment and modification); 11. MFI (Multifactorial interventions)

Refs A: references 1, 2, 8, 39 and 41 had ≥ 2 high-risk biases.

Refs B: references 5, 24, 32 and 45-49 had ≥5 unclear biases.

Refs C: references 1, 23 and 32 had a follow-up period of <6 months.

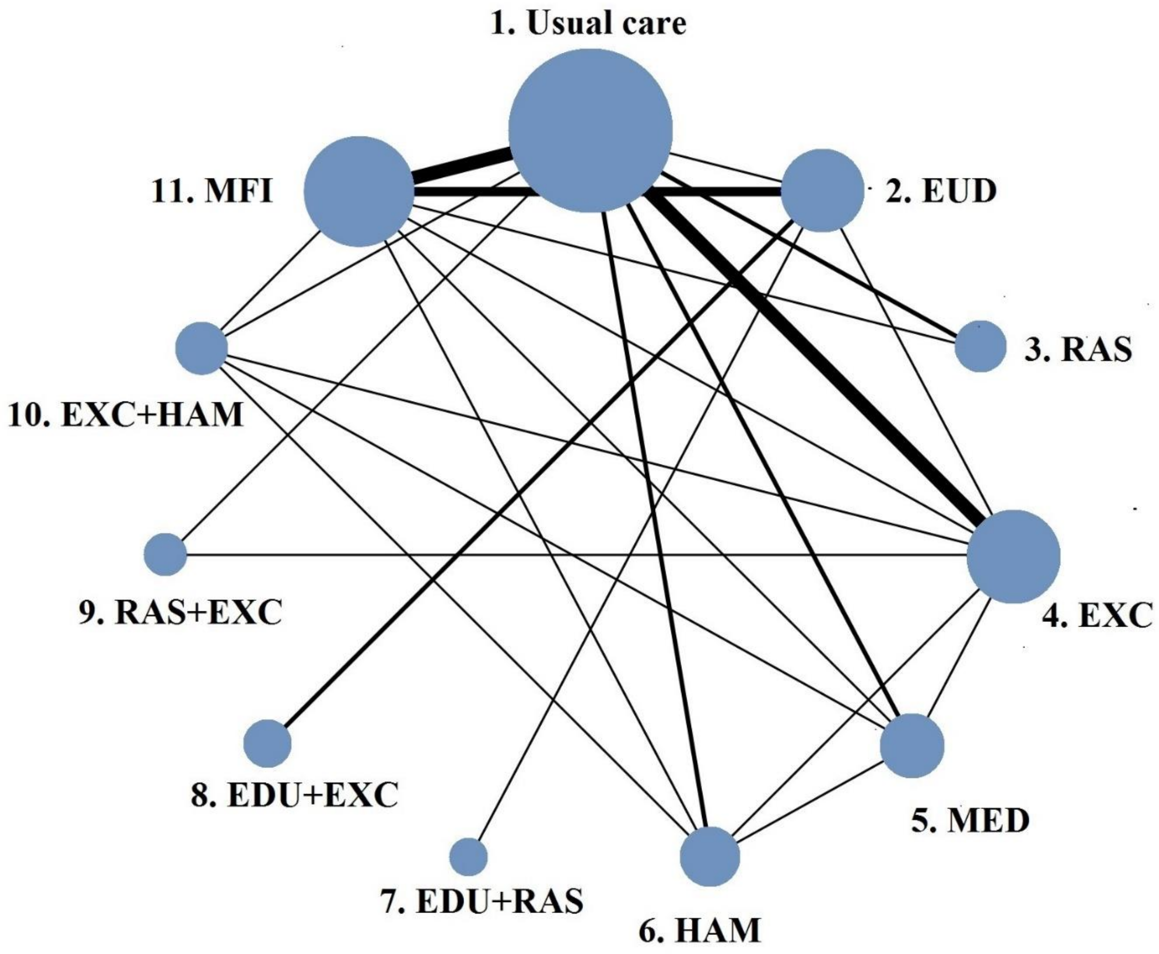

Figure S1: Network of interventions to prevent falls in the elderly

Labels of interventions: 1. Usual care (Namely without any specific fall intervention); 2. EDU (Education); 3. RAS (Risk assessment and suggestions); 4. EXC (Exercise); 5. MED (Medical care) 6. HAM (Hazard assessment and modification); 7. EDU+RAS (Education + risk assessment and suggestions); 8. EDU+EXC (Education + exercise); 9. RAS+EXC (Risk assessment and suggestions + exercise); 10. EXC+HAM (Exercise + hazard assessment and modification); 11. MFI (Multifactorial interventions).

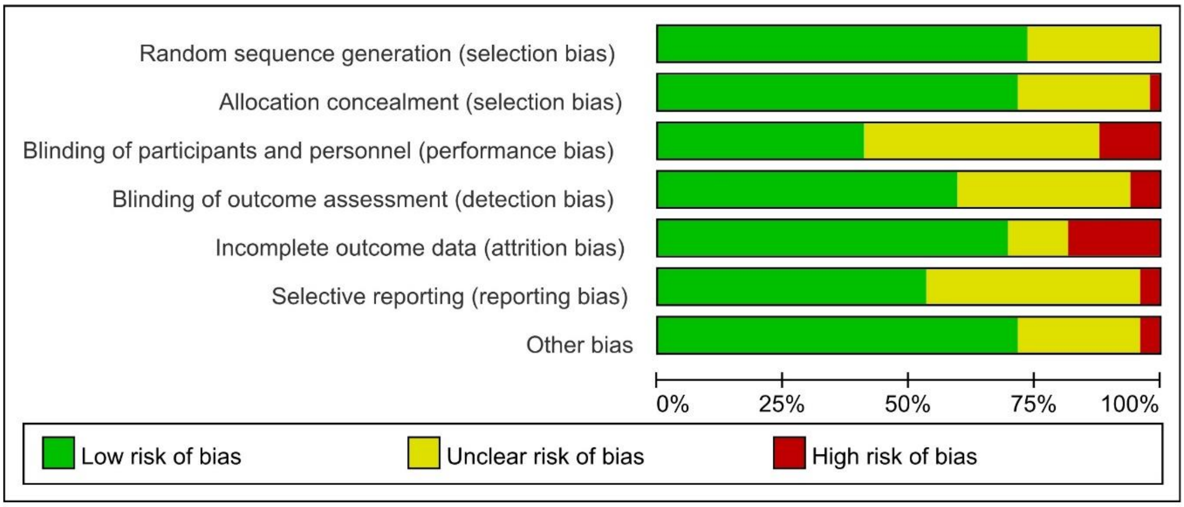

Figure S2: Judgments of risk for 7 items of bias in all 49 included studies

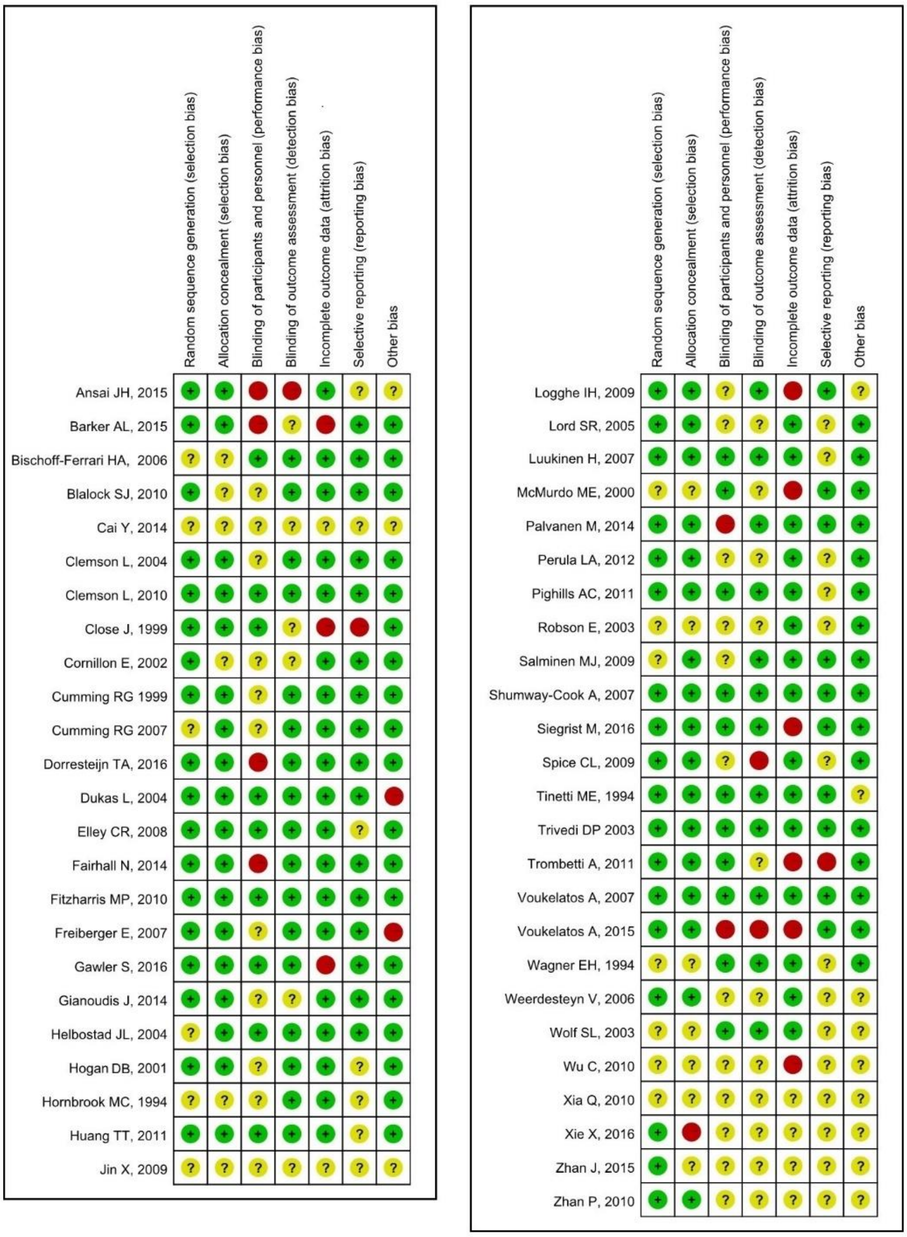

Figure S3: Risk summary of 49 studies included in NMA by item and article

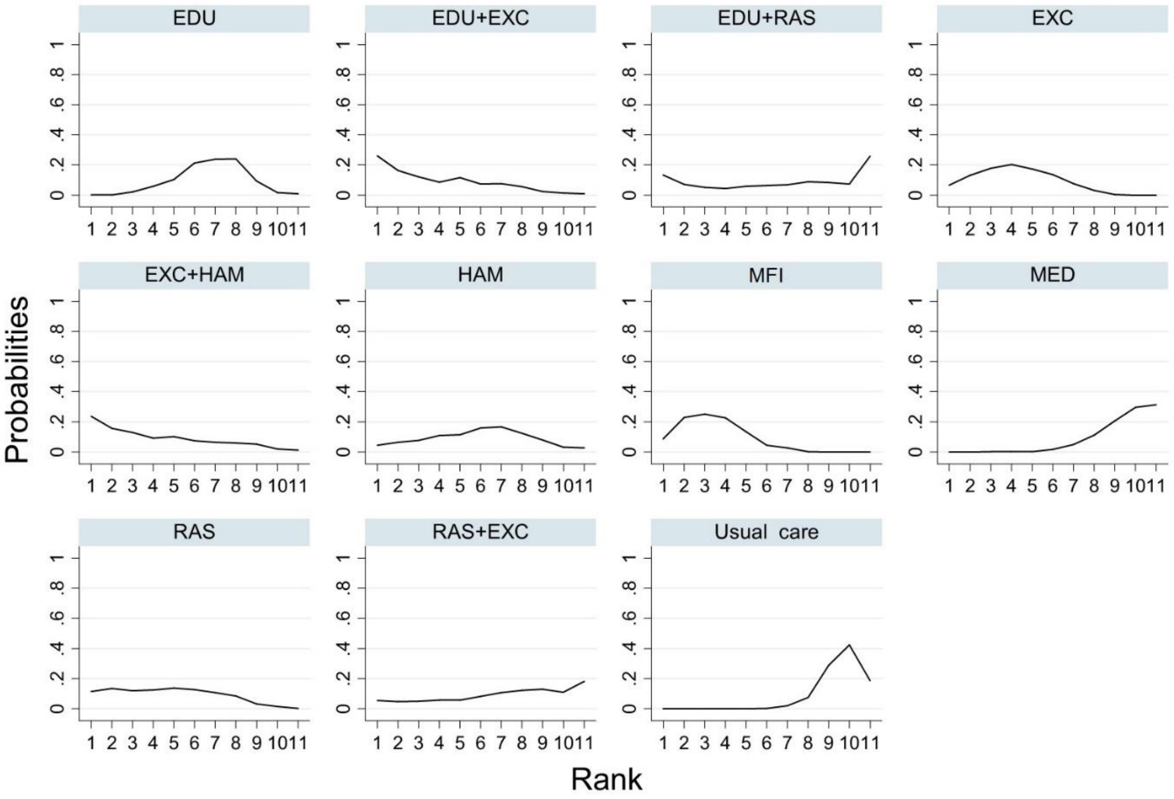

Figure S4: Ranking of intervention strategies based on probability of their effects on outcome of falls (Rankogram)

Labels of interventions: 1. Usual care (Namely without any specific fall intervention); 2. EDU (Education); 3. RAS (Risk assessment and suggestions); 4. EXC (Exercise); 5. MED (Medical care) 6. HAM (Hazard assessment and modification); 7. EDU+RAS (Education + risk assessment and suggestions); 8. EDU+EXC (Education + exercise); 9. RAS+EXC (Risk assessment and suggestions + exercise); 10. EXC+HAM (Exercise + hazard assessment and modification); 11. MFI (Multifactorial interventions).

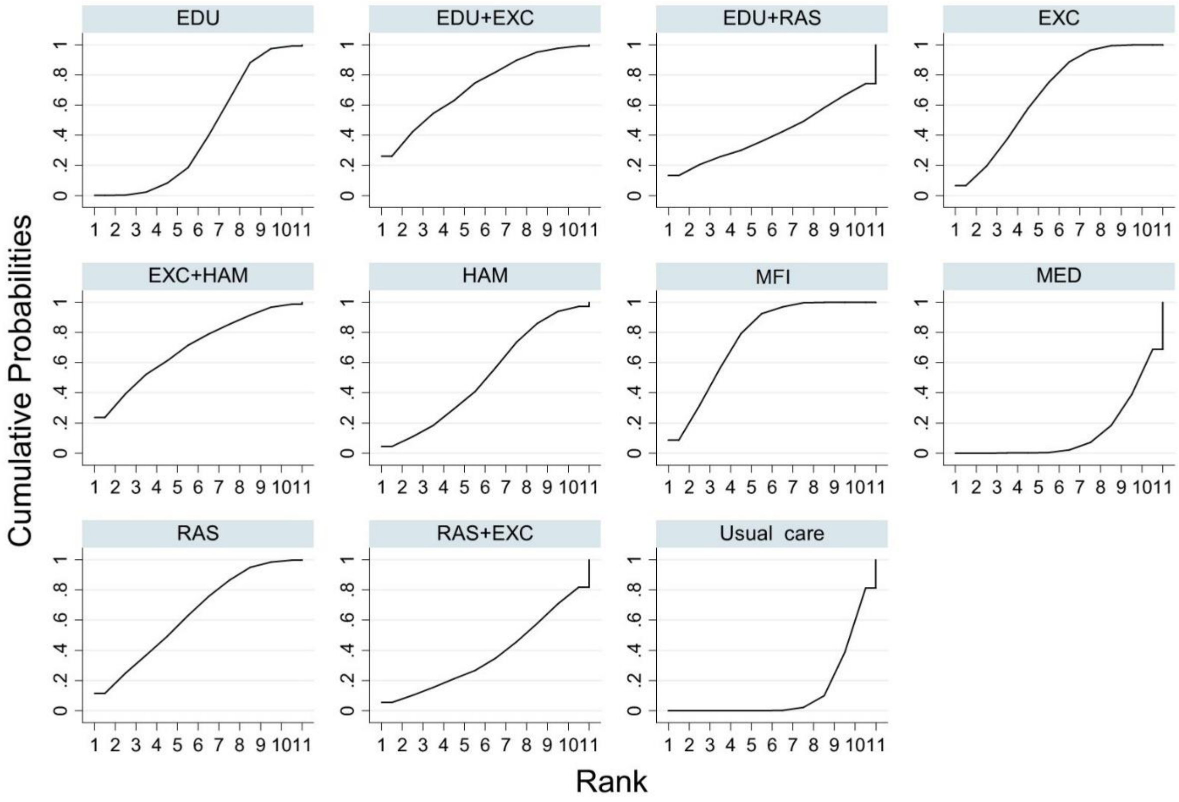

Figure S5: Surface under the cumulative ranking curve (SUCRA) probabilities diagram

Note: Surface under the cumulative ranking curve (SUCRA) probabilities is a percentage of the efficacy or safety of every intervention relative to an imaginary intervention that is always the best without uncertainty [50, 51]. Larger SUCRA scores indicate a more effective intervention.

Labels of interventions: 1. Usual care (Namely without any specific fall intervention); 2. EDU (Education); 3. RAS (Risk assessment and suggestions); 4. EXC (Exercise); 5. MED (Medical care) 6. HAM (Hazard assessment and modification); 7. EDU+RAS (Education + risk assessment and suggestions); 8. EDU+EXC (Education + exercise); 9. RAS+EXC (Risk assessment and suggestions + exercise); 10. EXC+HAM (Exercise + hazard assessment and modification); 11. MFI (Multifactorial interventions).

Study	Intervention Fallers	Intervention Total	Control Fallers	Control Total	Odds Ratio, 95%CI (CrI)
**1. RAS vs. Usual care**					
Close J, 1999	59	184	111	213	0.43 (0.29, 0.65)
Hogan DB, 2001	54	79	61	84	0.81 (0.41, 1.60)
Lord SR, 2005	25	202	37	201	0.63 (0.36, 1.09)
Wagner EH, 1994	93	317	177	607	1.01 (0.75, 1.36)
**Total**	**231**	**782**	**386**	**1105**	**0.69 (0.44, 1.07)**
Heterogeneity: Tau² = 0.14; Chi² = 11.09, df = 3 (P = 0.01); I² = 73%					
Network meta-anaylsis					0.69 (0.48, 0.96)
**2. EXC vs. Usual care**					
Ansai JH, 2015	15	45	13	23	0.38 (0.14, 1.08)
Clemson L, 2010	8	18	9	16	0.62 (0.16, 2.42)
Fitzharris MP, 2010	76	135	87	137	0.74 (0.46, 1.20)
Freiberger E, 2007	48	134	37	83	0.69 (0.40, 1.21)
Gawler S, 2016	203	457	131	252	0.74 (0.54, 1.00)
Siegrist M, 2016	73	222	70	156	0.60 (0.39, 0.92)
Trombetti A, 2011	19	66	32	68	0.45 (0.22, 0.93)
Voukelatos A, 2007	71	347	81	337	0.81 (0.57, 1.17)
Voukelatos A, 2015	54	159	68	180	0.85 (0.54, 1.32)
Weerdesteyn V, 2006	31	75	9	26	1.33 (0.53, 3.37)
Zhan P, 2010	7	50	18	50	0.29 (0.11, 0.78)
**Total**	**605**	**1708**	**555**	**1328**	**0.71 (0.60, 0.82)**
Heterogeneity: Chi² = 9.65, df = 10 (P = 0.47); I² = 0%					
Network meta-anaylsis					0.67 (0.52, 0.84)
**3. MED vs. Usual care**					
Bischoff-Ferrari HA, 2006	107	219	124	226	0.79 (0.54, 1.14)
Cumming RG 2007	201	309	153	307	1.87 (1.36, 2.59)
Dukas L, 2004	40	192	46	186	0.80 (0.49, 1.30)
Fitzharris MP, 2010	84	139	87	137	0.88 (0.54, 1.43)
Trivedi DP 2003	254	1345	261	1341	0.96 (0.79, 1.17)
**Total**	**686**	**2204**	**671**	**2197**	**1.02 (0.74, 1.40)**
Heterogeneity: Tau² = 0.10; Chi² = 17.07, df = 4 (P = 0.002); I² = 77%					
Network meta-anaylsis					1.02 (0.78, 1.34)
**4. HAM vs. Usual care**					
Cumming RG 1999	96	264	119	266	0.71 (0.50, 1.00)
Fitzharris MP, 2010	78	136	87	137	0.77 (0.48, 1.26)
Pighills AC, 2011	100	160	54	78	0.74 (0.42, 1.32)
**Total**	**274**	**1708**	**260**	**1328**	**0.73 (0.57, 0.94)**
Heterogeneity: Chi² = 0.09, df = 2 (P = 0.96); I² = 0%					
Network meta-anaylsis					0.76 (0.52, 1.11)
**5. MFI vs. Usual care**					
Cai Y, 2014	10	160	28	166	0.33 (0.15, 0.70)
Clemson L, 2004	82	157	89	153	0.79 (0.50, 1.23)
Cornillon E, 2002	39	143	48	150	0.80 (0.48, 1.32)
Elley CR, 2008	98	157	106	155	0.77 (0.48, 1.23)
Fairhall N, 2014	72	120	67	121	1.21 (0.72, 2.02)
Fitzharris MP, 2010	65	135	87	137	0.53 (0.33, 0.87)
Lord SR, 2005	28	194	37	201	0.75 (0.44, 1.28)
Robson E, 2003	41	235	55	236	0.70 (0.44, 1.09)
Spice CL, 2009	276	346	133	159	0.77 (0.47, 1.26)
Tinetti ME, 1994	52	147	68	144	0.61 (0.38, 0.98)
Wagner EH, 1994	199	635	177	607	1.11 (0.87, 1.41)
Wu C, 2010	11	87	16	103	0.79 (0.34, 1.80)
Xia Q, 2010	60	723	167	699	0.29 (0.21, 0.40)
**Total**	**1033**	**3239**	**1078**	**3031**	**0.68 (0.52, 0.89)**
Heterogeneity: Tau² = 0.18; Chi² = 54.78, df = 12 (P < 0.00001); I² = 78%					
Network meta-anaylsis					0.64 (0.53, 0.77)
**6. EDU+EXC vs. EDU**					
Barker AL, 2015	6	20	9	24	0.71 (0.20, 2.53)
Huang TT, 2011	3	56	16	120	0.37 (0.10, 1.32)
Logghe IH, 2009	58	138	59	131	0.88 (0.55, 1.43)
Shumway-Cook A, 2007	124	226	130	227	0.91 (0.63, 1.31)
**Total**	**191**	**440**	**214**	**502**	**0.84 (0.64, 1.11)**
Heterogeneity: Chi² = 1.88, df = 3 (P = 0.60); I² = 0%					
Network meta-anaylsis					0.81 (0.50, 1.21)
**7. MFI vs. EDU**					
Gianoudis J, 2014	29	81	25	81	1.25 (0.65, 2.40)
Hornbrook MC, 1994	628	1611	691	1571	0.81 (0.71, 0.94)
Jin X, 2009	44	849	63	829	0.66 (0.45, 0.99)
Palvanen M, 2014	296	611	349	653	0.82 (0.66, 1.02)
Perula LA, 2012	23	133	64	271	0.68 (0.40, 1.15)
Salminen MJ, 2009	140	292	131	297	1.17 (0.84, 1.61)
Xie X, 2016	8	140	21	140	0.34 (0.15, 0.80)
**Total**	**1168**	**3717**	**1344**	**3842**	**0.82 (0.74, 0.91)**
Heterogeneity: Chi² = 11.73, df = 6 (P = 0.07); I² = 49%					
Network meta-anaylsis					0.81 (0.64, 1.00)

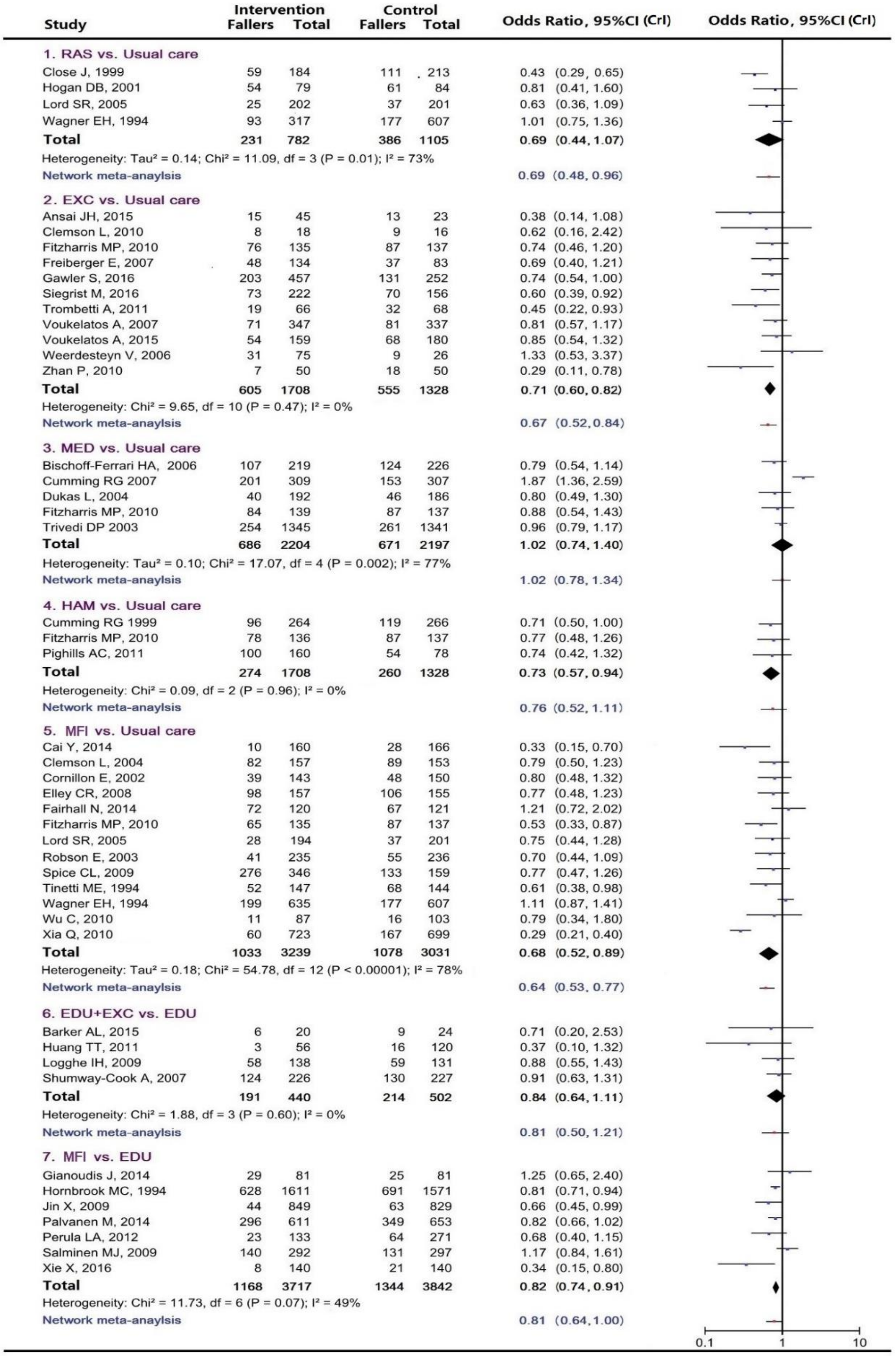

Figure S6: Pooled odds ratios for fall incidence by Bayesian network meta-

analysis and pairwise meta-analysis

Note: Only the comparisons reported in 3 studies or more were analyzed.

Labels of interventions: 1. Usual care (Namely without any specific fall intervention); 2. EDU (Education); 3. RAS (Risk assessment and suggestions); 4. EXC (Exercise); 5. MED (Medical care) 6. HAM (Hazard assessment and modification); 7. EDU+RAS (Education + risk assessment and suggestions); 8. EDU+EXC (Education + exercise); 9. RAS+EXC (Risk assessment and suggestions + exercise); 10. EXC+HAM (Exercise + hazard assessment and modification); 11. MFI (Multifactorial interventions).

95% CI: 95% confidence interval in meta-analysis, 95% CrI: 95% credible intervals in NMA.

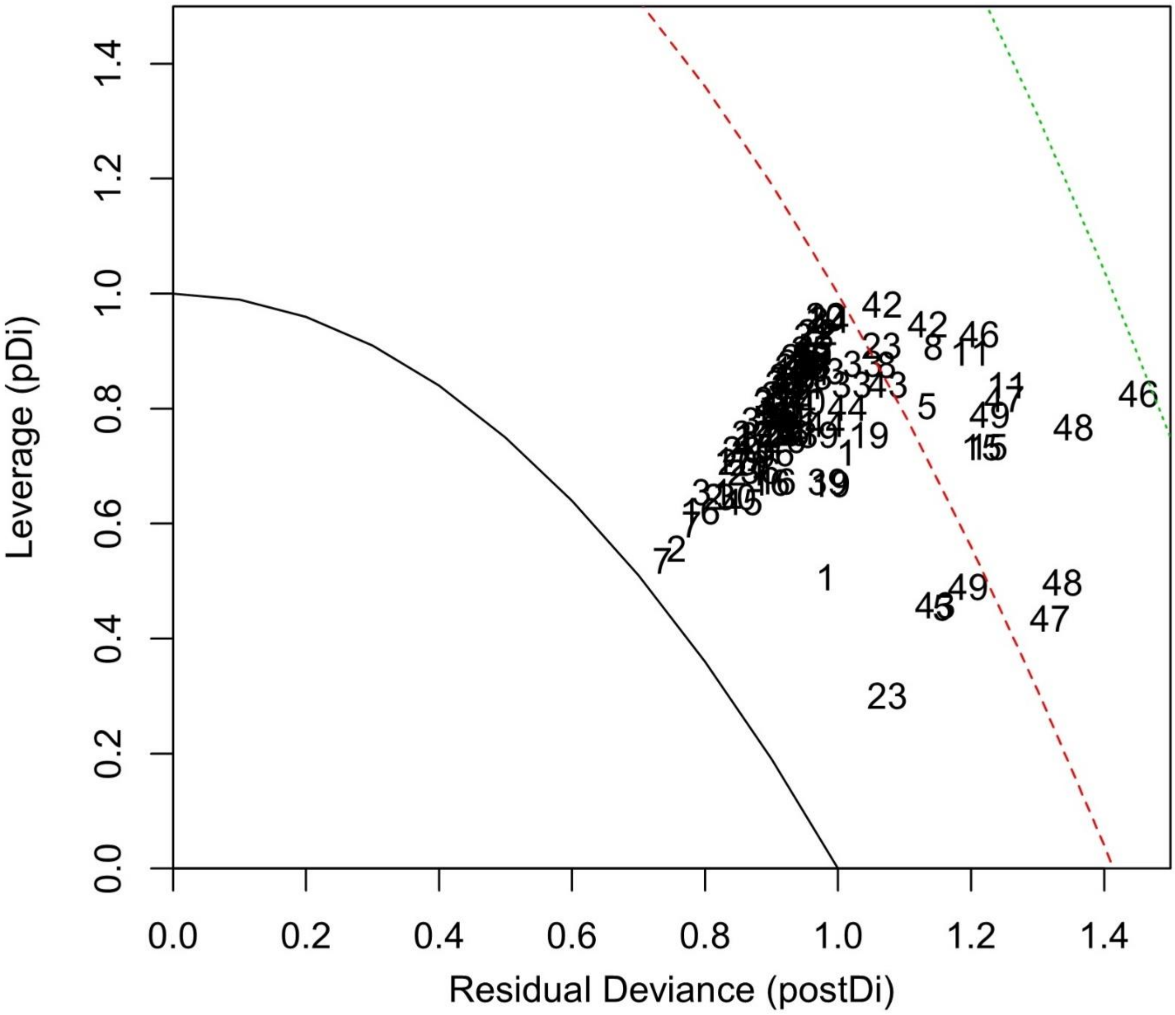

Figure S7: Benchmark effect diagram

Note: Residual deviance = 101.88, Data points =104 (Note that total residual deviance should approximate the number of data points for a good fit), Effective number of parameters= 64.04, DIC=165.92.

The diagram was calculated using code proposed by Georgia Salanti [52]. For an adequately fitting model, it will be approximately equal to the unconstrained data points [53]. From the diagram, we find that all arms of studies are in the x^2+y=3, meaning the model fit well.

Loop	IF	95%CI (truncated)	Loop-specific Heterogeneity(τ^2)
01-02-04	0.50	(0.00,1.28)	0.023
01-10-11	0.44	(0.00,1.94)	0.184
01-05-10	0.37	(0.00,1.79)	0.120
01-02-11	0.30	(0.00,1.04)	0.110
01-05-06	0.25	(0.00,1.26)	0.097
01-03-11	0.21	(0.00,1.00)	0.138
01-04-09	0.05	(0.00,1.09)	0.000
01-04-06	0.03	(0.00,0.62)	0.000

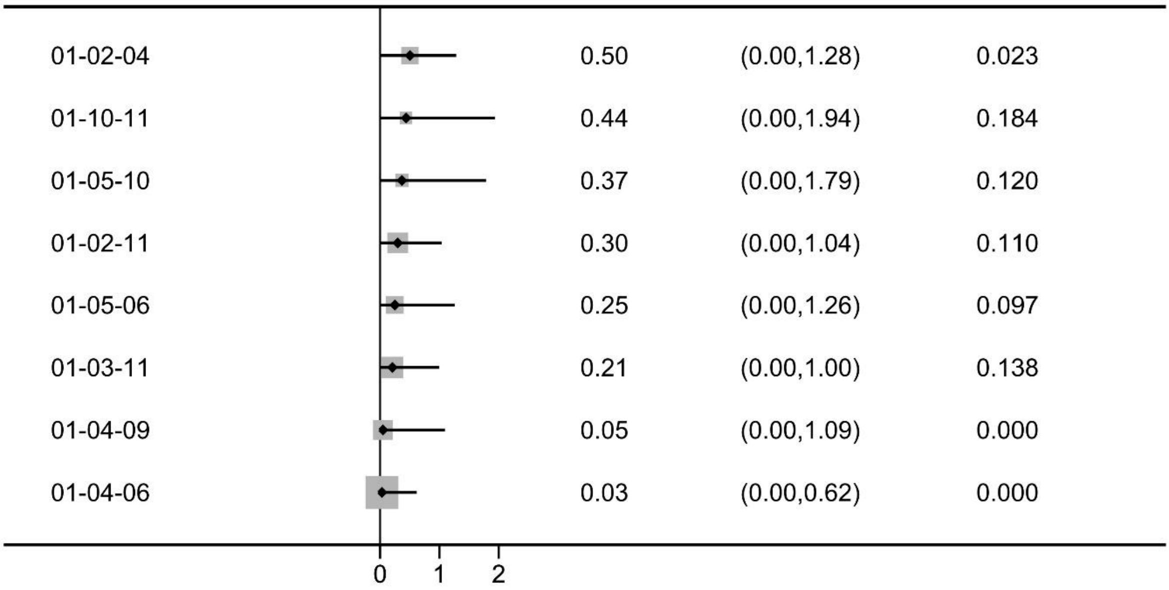

Figure S8: Inconsistency analysis using loop-specific heterogeneity estimate

Note: 8 triangular loops found.

IF*: inconsistency factor (IF), which is the absolute difference between direct and indirect estimates for one of the comparisons. Confidence intervals truncated at zero indicate no statistical significance of inconsistency [50].

Labels of interventions: 1. Usual care (Namely without any specific fall intervention); 2. EDU (Education); 3. RAS (Risk assessment and suggestions); 4. EXC (Exercise); 5. MED (Medical care) 6. HAM (Hazard assessment and modification); 7. EDU+RAS (Education + risk assessment and suggestions); 8. EDU+EXC (Education + exercise); 9. RAS+EXC (Risk assessment and suggestions + exercise); 10. EXC+HAM (Exercise + hazard assessment and modification); 11. MFI (Multifactorial interventions).

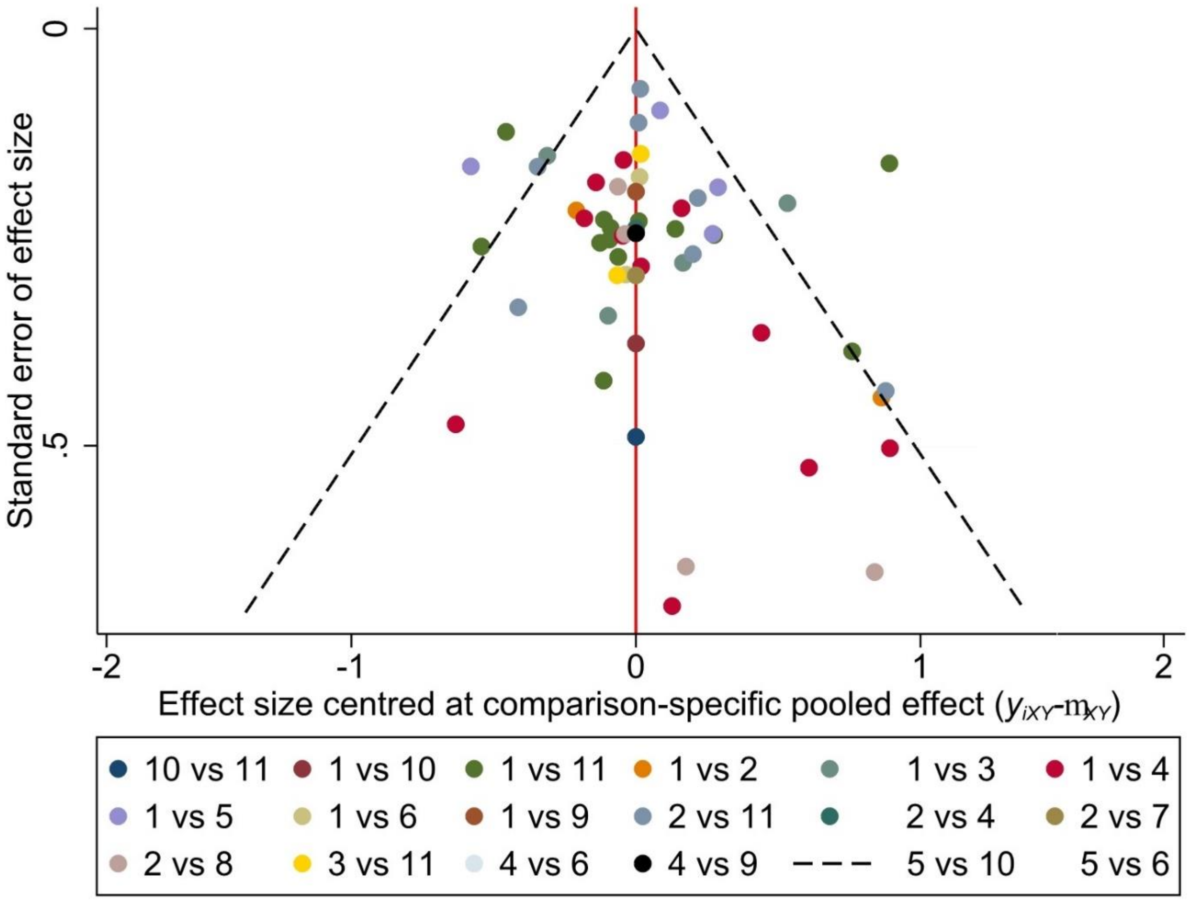

Figure S9: Comparison-adjusted funnel plot

Notes: The red full line represents the null hypothesis that the study-specific effect sizes do not differ from the respective comparison-specific pooled effect estimates. The two black dashed lines represent a 95% CI for the difference between study-specific effect sizes and comparison-specific summary estimates. Different colors correspond to different comparisons.

Labels of interventions: 1. Usual care (Namely without any specific fall intervention); 2. EDU (Education); 3. RAS (Risk assessment and suggestions); 4. EXC (Exercise); 5. MED (Medical care) 6. HAM (Hazard assessment and modification); 7. EDU+RAS (Education + risk assessment and suggestions); 8. EDU+EXC (Education + exercise); 9. RAS+EXC (Risk assessment and suggestions + exercise); 10. EXC+HAM (Exercise + hazard assessment and modification); 11. MFI (Multifactorial interventions).

References

1. Ansai, J.H.; Aurichio, T.R.; Gonçalves, R.; Rebelatto, J.R. Effects of two physical exercise protocols on physical performance related to falls in the oldest old: A randomized controlled trial. *Geriatr. Gerontol. Int*. **2016**, 16, 429-429; DOI: 10.1111/ggi.12497.
2. Barker, A.L.; Talevski, J.; Bohensky, M.A.; Brand, C.A.; Cameron, P.A.; Morello, R.T. Feasibility of Pilates exercise to decrease falls risk: A pilot randomized controlled trial in community-dwelling older people. *Clin. Rehabil*. **2016**, 30, 984-996; DOI: 10.1177/0269215515606197.
3. Bischoff-Ferrari, H.A.; Orav, E.J.; Dawson-Hughes, B. Effect of cholecalciferol plus calcium on falling in ambulatory older men and women: a 3-year randomized controlled trial. *Arch. Intern. Med*. **2006**, 166, 424-430; DOI: 10.1001/archinte.166.4.424.
4. Blalock, S.J.; Casteel, C.; Roth, M.T.; Ferreri, S.; Demby, K.B.; Shankar, V. Impact of enhanced pharmacologic care on the prevention of falls: A randomized controlled trial. *Am. J. Geriatr. Pharmacother*. **2010**, 8, 428-440; DOI: 10.1016/j.amjopharm.2010.09.002.
5. Cai, Y. Observation on the Effect of nursing intervention for fall among elderly adults in rural community. *Medical. Information*. **2014**, 27, 375; DOI: 10.3969/j.issn.1006-1959.2014.14.422.
6. Clemson, L.; Cumming, R.G.; Kendig, H.; Swann, M.; Heard, R.; Taylor, K. The Effectiveness of a Community-based program for reducing the incidence of falls in the elderly: A Randomized Trial. *J. Am. Geriatr. Soc*. **2004**, 52, 1487-94; DOI: 10.1111/j.1532-5415.2004.52411.x.
7. Clemson, L.; Singh, M.F.; Bundy, A.; Cumming, R.G.; Weissel, E.; Munro, J.; Manollaras, K.; Black, D. LiFE Pilot Study: A randomised trial of balance and strength training embedded in daily life activity to reduce falls in older adults. *Aust. Occup. Ther. J*. **2010**, 57, 42-50; DOI: 10.1111/j.1440-1630.2009.00848.x..
8. Close, J.; Ellis, M.; Hooper, R.; Glucksman, E.; Jackson, S.; Swift, C. Prevention of falls in the elderly trial (PROFET): a randomised controlled trial. *Lancet*. **1999**, 353, 93-97; DOI: 10.1186/1748-5908-7-48.
9. Cornillon, E.; Blanchon, M.A.; Ramboatsisetraina, P.; Braize, C.; Beauchet, O.; Dubost, V.; Blanc, P.; Gonthier, R. Effectiveness of falls prevention strategies for elderly subjects who live in the community with performance assessment of physical activities (before-after). *Ann. Readapt. Med. Phys*. **2002**, 45, 493-504.
10. Cumming, R.G.; Thomas, M.; Szonyi, G.; Salkeld, G.; O'Neill, E.; Westbury, C.; Frampton, G. Home visits by an occupational therapist for assessment and modification of environmental hazards: a randomized trial of falls prevention. *J. Am. Geriatr. Soc*. **1999**, 47, 1397-1402.
11. Cumming, R.G.; Ivers, R.; Clemson, L.; Cullen, J.; Hayes, M.F.; Tanzer, M.; Mitchell, P: Improving vision to prevent falls in frail older people: a randomized trial. *J. Am. Geriatr. Soc.* **2007**, 55, 175-181; DOI: 10.1111/j.1532-5415.2007.01046.x.
12. Dorresteijn, T.A.; Zijlstra, G.A.; Ambergen, A.W.; Delbaere, K.; Vlaeyen, J.W.; Kempen, G.I. Effectiveness of a home-based cognitive behavioral program to manage concerns about falls in community-dwelling, frail older people: results of a randomized controlled trial. *BMC. Geriatr*. **2016**, 16, 2; DOI: 10.1186/s12877-015-0177-y.
13. Dukas, L.; Bischoff, H.A.; Lindpaintner, L.S.; Schacht, E.; Birkner-Binder, D.; Damm, T.N.; Thalmann, B.; Stähelin, H.B. Alfacalcidol reduces the number of fallers in a community-dwelling elderly population with a minimum calcium intake of more than 500 mg daily. *J. Am. Geriatr. Soc*. **2004**, 52, 230-236.
14. Elley, C.R.; Robertson, M.C.; Garrett, S.; Kerse, N.M.; McKinlay, E.; Lawton, B.; Moriarty, H.; Moyes, S.A.; Campbell, A.J. Effectiveness of a falls-and-fracture nurse coordinator to reduce falls: a randomized, controlled trial of at-risk older adults. *J. Am. Geriatr. Soc*. **2008**, 56, 1383-1389; DOI: 10.1111/j.1532-5415.2008.01802.x.
15. Fairhall, N.; Sherrington, C.; Lord, S.R.; Kurrle, S.E.; Langron, C.; Lockwood, K.; Monaghan, N.; Aggar, C.; Cameron, I.D. Effect of a multifactorial, interdisciplinary intervention on risk factors for falls and fall rate in frail older people: A randomised controlled trial. *Age. Ageing*. **2014**, 43, 616-622; DOI: 10.1093/ageing/aft204.
16. Fitzharris, M.P.; Day, L.; Lord, S.R.; Gordon, I.; Fildes, B. The Whitehorse NoFalls trial: effects on fall rates and injurious fall rates. *Age. Ageing*. **2010**, 39, 728-733; DOI: 10.1093/ageing/afq109.

17. Freiberger, E, Menz, H.B, Abu-Omar, K, Rutten, A. Preventing falls in physically active community-dwelling older people: a comparison of two intervention techniques. *Gerontology*. **2007**, 53, 298-305; DOI: 10.1159/000103256.
18. Gawler, S.; Skelton, D.A.; Dinan-Young, S.; Masud, T.; Morris, R.W.; Griffin, M.; Kendrick, D.; Iliffe, S. Reducing falls among older people in general practice: The ProAct65+ exercise intervention trial. *Arch. Gerontol. Geriatr*. **2016**, 67, 46-54; DOI: 10.1016/j.archger.2016.06.019.
19. Gianoudis, J.; Bailey, C.A.; Ebeling, P.R.; Nowson, C.A.; Sanders, K.M.; Hill, K.; Daly, R.M. Effects of a targeted multimodal exercise program incorporating high-speed power training on falls and fracture risk factors in older adults: a community-based randomized controlled trial. *J. Bone. Miner. Res*. **2014**, 29, 182-191; DOI: 10.1002/jbmr.2014.
20. Helbostad, J.L.; Sletvold, O.; Moe-Nilssen, R. Effects of home exercises and group training on functional abilities in home-dwelling older persons with mobility and balance problems: a randomized study. *Aging. Clin. Exp. Res*. **2004**, 16, 113-121.
21. Hogan, D.B.; MacDonald, F.A.; Betts, J.; Bricker, S.; Ebly, E.M.; Delarue, B.; Fung, T.S.; Harbidge, C.; Hunter, M.; Maxwell, C.J.; Metcalf, B. A randomized controlled trial of a community-based consultation service to prevent falls. *CMAJ*. **2001**, 165, 537-543.
22. Hornbrook, M.C.; Stevens, V.J.; Wingfield, D.J.; Hollis, J.F.; Greenlick, M.R.; Ory, M.G. Preventing falls among community-dwelling older persons: results from a randomized trial. *Gerontologist*. **1994**, 34, 16-23.
23. Huang, T.T.; Yang, L.H.; Liu, C.Y. Reducing the fear of falling among community-dwelling elderly adults through cognitive-behavioural strategies and intense Tai Chi exercise: a randomized controlled trial. *J. Adv. Nurs*. **2011**, 67, 961-971; DOI: 10.1111/j.1365-2648.2010.05553.x.
24. Jin X.; Wang, S. The results analysis of intervention on fall among the elderly in Niujie area of Beijing. *Chinese. Journal. Of. Geriatrics*, **2009**, 28, 777-779; DOI: 10.3760/cma.j.issn.0254-9026.2009.09.024.
25. Logghe, I.H.; Zeeuwe, P.E.; Verhagen, A.P.; Wijnen-Sponselee, R.M.; Willemsen, S.P.; Bierma-Zeinstra, S.M.; van, Rossum, E.; Faber, M.J.; Koes, B.W. Lack of effect of Tai Chi Chuan in preventing falls in elderly people living at home: a randomized clinical trial. *J. Am. Geriatr. Soc*. **2009**, 57, 70-75; DOI: 10.1111/j.1532-5415.2008.02064.x.
26. Lord, S.R.; Tiedemann, A.; Chapman, K.; Munro, B.; Murray, S.M.; Gerontology. M.; Ther, G.R.; Sherrington, C. The effect of an individualized fall prevention program on fall risk and falls in older people: a randomized, controlled trial. *J. Am. Geriatr. Soc*. **2005**, 53, 1296-1304; DOI: 10.1111/j.1532-5415.2005.53425.x.
27. Luukinen, H.; Lehtola, S.; Jokelainen, J.; Väänänen-Sainio, R.; Lotvonen, S.; Koistinen, P. Pragmatic exercise-oriented prevention of falls among the elderly: a population-based, randomized, controlled trial. *Prev. Med*. **2007**, 44, 265-271; DOI: 10.1016/j.ypmed.2006.09.011.
28. McMurdo, M.E.; Millar,. AM.; Daly, F. A randomized controlled trial of fall prevention strategies in old peoples' homes. *Gerontology*. **2000**, 46, 83-87; DOI: 10.1159/000022139.
29. Palvanen, M.; Kannus, P.; Piirtola, M.; Niemi, S.; Parkkari, J.; Järvinen, M. Effectiveness of the Chaos Falls Clinic in preventing falls and injuries of home-dwelling older adults: a randomised controlled trial. *Injury*. **2014**, 45, 265-271; DOI: 10.1016/j.injury.2013.03.010.
30. Pérula, L.A.; Varas-Fabra, F.; Rodríguez, V.; Ruiz-Moral, R.; Fernández, J.A.; González, J.; Pérula, C.J.; Roldán, A.M.; de, Dios, C. Effectiveness of a multifactorial intervention program to reduce falls incidence among community-living older adults: a randomized controlled trial. *Arch. Phys. Med. Rehabil*. **2012**, 93, 1677-1684, DOI: 10.1016/j.apmr.2012.03.035.
31. Pighills, A.C.; Torgerson, D.J.; Sheldon, T.A.; Drummond, A.E. Environmental assessment and modification to prevent falls in older people. *J. Am. Geriatr. Soc*. **2011**, 59, 26-33; DOI: 10.1111/j.1532-5415.2010.03221.x.
32. Robson, E.; Edwards, J.; Gallagher, E.; Baker, D. Steady as you go (SAYGO): a falls-prevention program for seniors living in the community. *Can. J. Aging*. **2003**, 22, 207-216; DOI: 10.1017/S0714980800004529.
33. Salminen, M.J.; Vahlberg, T.J.; Salonoja, M.T.; Aarnio, P.T.; Kivelä, S.L. Effect of a risk-based multifactorial fall prevention program on the incidence of falls. *J. Am. Geriatr. Soc*. **2009**, 57, 612-619; DOI: 10.1111/j.1532-5415.2009.02176.x.

34. Shumway-Cook, A.; Silver, I.F.; Lemier, M.; York, S.; Cummings, P.; Koepsell, T.D. Effectiveness of a community-based multifactorial intervention on falls and fall risk factors in community-living older adults: a randomized controlled trial. *J. Gerontol. A. Biol. Sci. Med. Sci*. **2007**, 6, 1420-1427.
35. Siegrist, M.; Freiberger, E.; Geilhof, B.; Salb, J.; Hentschke, C.; Landendoerfer, P.; Linde, K.; Halle, M.; Blank, W.A. Fall Prevention in a Primary Care Setting. *Dtsch. Arztebl. Int*. **2016**, 113, 365-372; DOI: 10.3238/arztebl.2016.0365.
36. Spice, C.L.; Morotti, W.; George, S.; Dent, T.H.; Rose, J.; Harris, S.; Gordon, C.J. The Winchester falls project: a randomised controlled trial of secondary prevention of falls in older people. *Age. Ageing*. **2009**, 38, 33-40; DOI: 10.1093/ageing/afn192.
37. Tinetti, M.E.; Baker, D.I.; Mcavay, G.; Claus, E.B.; Garrett, P.; Gottschalk, M.; Koch, M.L.; Trainor, K.; Horwitz, R.I. A multifactorial intervention to reduce the risk of falling among elderly people living in the community. *N. Engl. J. Med*. **1994**, 331, 821-827; DOI: 10.1056/NEJM199409293311301.
38. Trivedi, D.P.; Doll, R.; Khaw, K.T. Effect of four monthly oral vitamin D3 (cholecalciferol) supplementation on fractures and mortality in men and women living in the community: randomised double blind controlled trial. *BMJ*. **2003**, 326, 469; DOI: 10.1136/bmj.326.7387.469.
39. Trombetti, A.; Hars, M.; Herrmann, F.R.; Kressig, R.W.; Ferrari, S.; Rizzoli, R. Effect of music-based multitask training on gait, balance, and fall risk in elderly people: a randomized controlled trial. *Arch. Intern. Med*. **2011**, 171, 525-533; DOI: 10.1001/archinternmed.2010.446.
40. Voukelatos, A.; Cumming, R.G.; Lord, S.R.; Rissel, C. A randomized, controlled trial of tai chi for the prevention of falls: the Central Sydney tai chi trial. *J. Am. Geriatr. Soc*. **2007**, 55, 1185-1191; DOI: 10.1111/j.1532-5415.2007.01244.x.
41. Voukelatos, A.; Merom, D.; Sherrington, C.; Rissel, C.; Cumming, R.G.; Lord, S.R. The impact of a home-based walking programme on falls in older people: The Easy Steps randomised controlled trial. *Age. Ageing*. **2015**, 44, 377-383; DOI: 10.1093/ageing/afu186.
42. Wagner, E.H.; LaCroix, A.Z.; Grothaus, L.; Leveille, S.G.; Hecht, J.A.; Artz, K.; Odle, K.; Buchner, D.M. Preventing disability and falls in older adults: a population-based randomized trial. *Am. J. Public. Health*. **1994**, 84, 1800-1806.
43. Weerdesteyn, V.; Rijken, H.; Geurts, A.C.; Smits-Engelsman, B.C.; Mulder, T.; Duysens, J. A five-week exercise program can reduce falls and improve obstacle avoidance in the elderly. *Gerontology*. **2006**, 52, 131-141; DOI: 10.1159/000091822.
44. Wolf, S.L.; Sattin, R.W.; Kutne,r M.; O'Grady, M.; Greenspan, A.I.; Gregor, R.J. Intense tai chi exercise training and fall occurrences in older, transitionally frail adults: a randomized, controlled trial. *J. Am. Geriatr. Soc*. **2003**, 51, 1693-1701.
45. Wu, C.; Wang, K.; He, Y.; Wang, Y.; Li, M.; Jin, P.; Wu, Y.; Hu, Y. A randomized controlled trial to prevent community elder falling and related factor analysis. *Chinese. Journal. Of. Rehabilitation. Medicine*. **2010**, 25, 986-988; DOI: 10.3969/j.issn.1001-1242.2010.10.015.
46. Xia, Q.; Jiang, Y.; Niu, C.; Xia, Z. Study on the effect of comprehensive intervention for fall among elderly adults in community. *Chinese. Journal. Of. Prevention. And. Control. Of. Chronic. Diseases*. **2010**, 18, 515-517.
47. Xie, X.; Huang, Z.; Zhang, H. Application of Community Intervention Combined Health Education in Preventing Falls in older people. *Chinese. Journal. Of. Ethno-medicine. And. Ethno-pharmacy*. **2016**, 25, 161-162.
48. Zhan, J. Observation on the effect of family prevention and prevention of falls in the elderly. *Medical. Information*. **2015**, 28, 226; DOI: 10.3969/j.issn.1006-1959.2015.37.325.
49. Zhan, P.; Liu, J. Application of CSPDCA mode in the design of exercises for the prevention of falls in the elderly. *Practical. Clinical. Medicine*. **2010**, 11, 98-99; DOI: 10.3969/j.issn.1009-8194.2010.12.059.
50. Chaimani, A.; Higgins, J.P.; Mavridis, D.; Spyridonos, P.; Salanti, G. Graphical Tools for Network Meta-Analysis in STATA. *PloS. One*. **2013**, 8, e76654; DOI: 10.1371/journal.pone.0076654.
51. Salanti, G.; Ades, A.E.; Ioannidis, J.P. Graphical methods and numerical summaries for presenting results from multiple-treatment meta-analysis: an overview and tutorial. *J. Clin. Epidemiol.* **2011**, 64, 163-171; DOI: 10.1016/j.jclinepi.2010.03.016.
52. Measure the fit of the model in R. Available online: http://www.mtm.uoi.gr/images/5.BayesDiagnoshelp.pdf (accessed on 12 Aug 2017).

53. Spiegelhalter, D.J.; Best, N.G.; Carlin, B.K.; van, der, Linde, A. Bayesian measures of model complexity and fit. *J. R. Statist. Soc. B*. **2002**, 64, 583–639; DOI: 10.1111/1467-9868.00353.
